# Emotion Regulation Difficulties Account for the Associations Between Childhood Maltreatment and Perceived Stress in Young Adulthood

**DOI:** 10.1007/s40653-026-00946-8

**Published:** 2026-07-21

**Authors:** Gaia Lapomarda, Beatrice Frajo-Apor, Gerald Autengruber, Alex Hofer, Carmen Morawetz

**Affiliations:** 1 Department of Psychology, Universität Innsbruck, Innsbruck, Austria; 2 Department of Psychiatry, Psychotherapy, Psychosomatics and Medical Psychology, Division of Psychiatry I, Innsbruck Medical University, Innsbruck, Austria

**Keywords:** Childhood maltreatment, Emotion dysregulation, Perceived stress, Associational variable analysis

## Abstract

Childhood exposure to traumatic events can have enduring negative effects, particularly on stress perception and overall well-being. Emotion dysregulation, which refers to difficulties in managing emotional responses, is frequently observed among individuals with a history of interpersonal trauma and has been proposed as a construct linking childhood maltreatment to heightened stress in adulthood. Using an Associational Variable Analysis (AVA), this study examined whether emotion regulation difficulties accounted for shared variance between childhood maltreatment and perceived stress in adulthood. A sample of 351 university students completed self-report assessments of childhood maltreatment (CTQ), emotion regulation difficulties (DERS), and perceived stress (PSS-10). At the total-score level, emotion regulation difficulties significantly accounted for the association between emotional neglect and perceived stress, whereas emotional abuse and sexual abuse maintained direct associations with stress. At the subscale level, specific emotion regulation difficulties accounted for the associations of emotional abuse and emotional neglect with both perceived helplessness and perceived self-efficacy, with limited access to emotion regulation strategies and difficulties engaging in goal-directed behavior emerging as the most relevant regulatory dimensions. These findings highlight emotion regulation difficulties as an important construct linking childhood emotional maltreatment with perceived stress in adulthood, while emphasizing the need for longitudinal designs before causal conclusions can be drawn.

**Supplementary Information:**

The online version contains supplementary material available at https://doi.org/10.1007/s40653-026-00946-8.

## Introduction

Childhood maltreatments refer to a variety of traumatic events that occur during childhood, including emotional and physical abuse and neglect (Cicchetti & Toth, 2005; World Health Organization, 2020). Meta-analytic evidence indicates that approximately 36.3% and 22.6% of individuals self-report experiences of childhood emotional and physical abuse, respectively (Stoltenborgh et al., 2012, 2013b), while 18.4% and 16.3% report emotional and physical neglect (Stoltenborgh et al. 2013a, b), and 12.7% report childhood sexual abuse (Stoltenborgh et al., 2011). Traumatic experiences during childhood have far-reaching consequences (Cicchetti & Toth, 1995), extending beyond immediate harm to include long-term impacts on mental health and overall well-being (Jennissen et al., 2016; World Health Organization, 1999, 2020), as well as disrupted ability to regulate emotions and form stable relationships (Heleniak et al., 2016; McLaughlin & Lambert, 2017). The “Report of the Consultation on Child Abuse Prevention” provides a comprehensive overview of the various types of maltreatment (World Health Organization, 1999), which encompasses Physical Abuse, Emotional Abuse, Sexual Abuse, and Physical and Emotional Neglect. Physical abuse is the actual or potential physical harm resulting from an action or lack of action within a caregiver’s control (Stoltenborgh et al., 2015). Emotional abuse occurs when there is a failure to provide a developmentally appropriate and supportive environment, which is crucial to developing a stable and full range of emotional and social competencies (Stoltenborgh et al., 2015). It also includes actions directed at children that cause harm or are likely to harm their mental or social development, such as denigrating, threatening, intimidating, discriminating, ridiculing, or using other non-physical forms of hostile or rejecting behavior (World Health Organization, 1999). Sexual abuse occurs when children are involved in sexual activity they cannot comprehend, give informed consent for, or are not developmentally prepared to engage in (Stoltenborgh et al., 2015). Finally, the description of Neglect - both physical and emotional - is characterized by a caregiver’s failure to provide for children’s development in areas such as health, education, emotional well-being, nutrition, shelter, and safe living conditions, taking into account the caregiver’s available resources (Stoltenborgh et al., 2015). Increasing awareness of typical child development and the harmful health effects of maltreatment underscores the urgency of addressing this issue.

Individuals with a history of childhood maltreatment show a diminished ability to cope with aversive affective states and experience higher levels of perceived stress (Hager & Runtz, 2012; Hong et al., 2018; Vranceanu et al., 2007). An expanding body of research suggests that early life maltreatment is associated with long-term increases in sensitivity to stressful life events, increasing the likelihood of psychopathology and unfavorable health consequences (Scorza et al., 2022). Neurobiological evidence further indicates that maltreated individuals have alterations in stress-susceptible brain regions, hypothalamic-pituitary-adrenal response, and inflammatory marker levels (Nemeroff, 2016; Tarullo & Gunnar, 2006; Teicher et al., 2022). Given the high prevalence and profound impacts of childhood maltreatment, understanding the mechanisms underlying these adverse outcomes is essential (Jennissen et al., 2016).

The long-term impacts of childhood maltreatment on stress sensitivity and neurobiological functioning are deeply interconnected with the development of emotional and behavioral difficulties (Dadomo et al., 2022; Grecucci et al., 2023). Children who experience maltreatment face a heightened risk of developing reactive aggression and deficits in regulating emotions (Dvir et al., 2014). Moreover, adults with a history of childhood maltreatment tend to detect less positive emotions and be faster in recognizing negative ones (Bérubé et al., 2023) and show difficulties in many forms of emotion regulation (Heleniak et al., 2016; McLaughlin & Lambert, 2017). Emotion regulation is defined as the processes of monitoring, evaluating, and modifying emotional reactions, particularly their intensity and duration, to achieve specific goals (Gross, 2015; Morawetz et al., 2017, 2020). It involves six dimensions, such as emotional awareness, acceptance, and clarity, the ability to inhibit prepotent responses, engage regulation strategies, and activate goal-directed behavior (Cole et al., 2019). During childhood, responsive parenting and peer interactions play a crucial role in fostering emotion regulation skills. Consequently, experiencing abuse and neglect as a child can hinder the development of proper emotional and interpersonal abilities (Dadomo et al., 2022; Dvir et al., 2014; Grecucci et al., 2023). Emotion dysregulation - often a result of early adversity - is commonly conceptualized as a multidimensional construct that involves the disruption of one or more of the emotion regulation dimensions, leading to (1) a lack of awareness, understanding, and acceptance of emotions, (2) limited access to adaptive strategies for modulating the intensity and/or duration of emotional responses, (3) an unwillingness to experience emotional distress in the pursuit of desired goals, and (4) an inability to control behaviors while experiencing emotional distress (Gratz et al., 2009; Gratz & Roemer, 2004).

Emotion regulation plays a crucial role in how individuals evaluate and respond to stressful events, shaping their emotional reactions and expressions (Wang & Saudino, 2011). This developmental link between emotion regulation and stress is particularly important, as children’s capacity to manage stress typically improves as they develop better emotional regulation skills (Wang & Saudino, 2011). Given the centrality of emotion regulation in effectively coping with stress, it is particularly concerning that difficulties in emotion regulation can exacerbate pre-existing vulnerabilities, such as impulsivity or behavioral inhibition, thereby contributing to more severe symptoms across various mental disorders (Beauchaine, 2015; Grecucci et al., 2022; Morawetz et al., 2024).

Thus, emotion dysregulation has been proposed as an important process associated with the relationship between childhood maltreatment and a range of long-term cognitive, psychological, and health outcomes (Beauchaine, 2015; Sheppes et al., 2015; Strathearn et al., 2020). Understanding these connections is crucial for developing effective interventions aimed at mitigating the enduring effects of childhood maltreatment. However, while emotion dysregulation has been identified as a mediator between childhood maltreatment and psychopathologies (Jennissen et al., 2016), the broader relationship between various forms of childhood maltreatment, emotion regulation difficulties, and perceived stress remains underexplored. Gaining a deeper understanding of this association is critical for developing targeted interventions that address the complex interplay of factors contributing to the long-term effects of childhood maltreatment.

Interestingly, the impact of childhood maltreatment on psychological functioning (Gallo et al., 2018; Godinet et al., 2014) and on the development of psychopathology (Prachason et al., 2024) seems to be different among females and males. As previously mentioned, emotion regulation has been identified as an important factor associated with the long-term consequences of childhood maltreatment (Kerig, 2020), and difficulties in this process are more common among females than males (Herd & Kim-Spoon, 2021; Vanhalst et al., 2018). In fact, the former tend to show a stronger inclination toward self-blame, ruminating, and catastrophizing following an adverse experience. Sex differences also emerge in perceiving stress and coping (Graves et al., 2021), as well as in the physiological stress responses (i.e., cortisol reactivity) (de Veld et al., 2012), with females showing higher levels of stress and stronger cortisol responses than males. Although studies on a variety of topics have highlighted the importance of sex-specific analyses (Glaesmer et al., 2011; Hong et al., 2018; Topitzes et al., 2012), mixed evidence persists, warranting further investigation (Prevoo et al., 2017).

Previous studies have examined stress responses as a risk factor for psychopathology, yet relatively little is known about how maltreatment relates to stress perception and regulation strategies that individuals acquire and use. To fill this gap, our study examines the associations between early traumatic experiences and perceived stress in adulthood, with a focus on the role of emotion dysregulation as an associational variable. Although emotion dysregulation has frequently been examined as a mediator of the effects of childhood maltreatment, most available evidence is based on cross-sectional data, which cannot establish temporal ordering or causal pathways (Maxwell & Cole, 2007; O’Laughlin et al., 2018; Weems, 2025). Therefore, alternative explanations can be considered, such as the possibility that perceived stress and emotion regulation difficulties reflect shared variance arising from common antecedents. To address these limitations, we followed the Associational Variable Analysis (AVA) framework proposed by Weems (2025), which is recommended when data can establish shared variance among variables but cannot provide temporal or causal mediation. Thus, we investigated whether difficulties in emotion regulation account for the shared variance between different traumatic experiences, such as abuse and neglect, and adult perceived stress, defined by two dimensions: perceived helplessness and self-efficacy. We hypothesized that difficulties in regulating emotions would account for a significant portion of the association between early-life traumatic experiences and perceived stress. Through this study, we aim to provide a deeper understanding of the associational patterns linking childhood maltreatment to stress in young adulthood.

## Method

### Sample

A sample of 351 participants (260 females, 91 males; mean age 23.13 ± 3.66) completed self-report measures of childhood maltreatment (CTQ), emotion regulation difficulties (DERS), and perceived stress (PSS-10), using LimeSurvey (https://www.limesurvey.org/). Participants were all students at the University of Innsbruck and the Medical University of Innsbruck, recruited through advertisements.

### Measures

#### Childhood Trauma Questionnaire - Short Form (CTQ)

Childhood maltreatment is here defined as exposure to traumatic experiences during childhood. Participants completed the German version of the Childhood Trauma Questionnaire - Short Form (Bernstein et al., 2003; Klinitzke et al., 2012). The CTQ is a retrospective, 28-item self-report scale that assesses the frequency of maltreatment experiences during childhood, including abuse and neglect. The items are rated on a 5-point Likert scale ranging from 1 (never true) to 5 (very often true), and explore five dimensions of maltreatment: (1) Emotional Abuse - verbal assaults or any humiliating or demeaning behavior directed at a child by an adult (e.g., “People in my family said hurtful or insulting things to me”), (2) Physical Abuse - bodily assaults on a child by an adult that created a danger of or led to harm (e.g., “People in my family hit me so hard that it left me with bruises or marks”), (3) Sexual Abuse - sexual contact or conduct between a child and an adult (e.g., “Someone tried to touch me in a sexual way, or tried to make me touch them”), (4) Emotional Neglect - failure of caretakers to meet children’s basic emotional and psychological needs, including love, belonging, nurturance, and support (e.g., “People in my family looked out for each other”, reversed-scored), and (5) Physical Neglect - failure of caretakers to provide for a child’s basic physical needs, including food, shelter, clothing, safety, and health care (e.g., “I had to wear dirty clothes”). Cronbach’s alpha for CTQ is α = 0.91.

#### Difficulties in Emotion Regulation Scale (DERS)

Participants completed the German version of the Difficulties in Emotion Regulation Scale (Ehring et al., 2013). The DERS is a 36-item self-report scale measuring difficulties within the following dimensions of emotion regulation: awareness and understanding of emotions, acceptance of emotions, the ability to engage in goal-directed behavior and refrain from impulsive behavior when experiencing negative emotions, and access to emotion regulation strategies perceived as effective for feeling better (Gratz & Roemer, 2004). Specifically, six subscales are defined: (1) Non-acceptance of emotional responses (e.g., “When I’m upset, I become angry with myself for feeling that way”), (2) Difficulty engaging in goal-directed behaviors (e.g., “When I’m upset, I have difficulty focusing on other things”), (3) Impulse control difficulties (e.g., “When I’m upset, I become out of control”), (4) Lack of emotional awareness (e.g., “When I’m upset, I acknowledge my emotions”, reversed-scored), (5) Limited access to emotion regulation strategies (e.g., “When I’m upset, I believe that I will remain that way for a long time”), and (6) Lack of emotional clarity (e.g., “I am confused about how I feel”). The items are rated on a 5-point Likert scale ranging from 1 (almost never) to 5 (almost always). Cronbach’s alpha for DERS is α = 0.95. Subscale alphas are: Non-acceptance of emotional responses, α = 0.88; Difficulty engaging in goal-directed behaviors, α = 0.90; Impulse control difficulties, α = 0.89; Lack of emotional awareness, α = 0.83; Limited access to emotion regulation strategies, α = 0.91; Lack of emotional clarity, α = 0.90.

#### Perceived Stress Scale (PSS-10)

Participants completed the German version of the Perceived Stress Scale (Schneider et al., 2020), a 10-item self-report scale that assesses the degree to which, in the last month, individuals perceived situations in their lives as uncontrollable, unpredictable, and overloaded. It is represented by two subscales: Perceived Helplessness - measuring individuals’ perceived difficulty in controlling circumstances or emotions (e.g., “In the last month, how often have you been upset because of something that happened unexpectedly?”), and Perceived Self-Efficacy - measuring individuals’ perceived ability to handle problems (e.g., “In the last month, how often have you felt confident about your ability to handle your personal problems?”). Items are rated on a 5-point scale from 1 (never) to 5 (very often). Cronbach’s alpha for PSS is α = 0.88.

### Statistical Analysis

All data were entered and analyzed in R (R Core Team, 2022). Following an initial exploration of descriptive statistics for the sample, we conducted preliminary analyses to examine potential sex and age differences in the variables of interest. Because the variables did not follow a normal distribution, non-parametric analyses were computed (i.e., Mann-Whitney U tests adjusted with the Benjamini-Hochberg method). Given the non-experimental cross-sectional design, the main models were framed according to the Associational Variable Analysis (AVA) framework proposed by Weems (2025), which is recommended when data can establish shared variance among variables but cannot establish temporal or causal mediation. Accordingly, emotion regulation difficulties were treated as associational variables that may account for shared variance between childhood maltreatment and perceived stress.

AVA proceeded in four steps. Step 1 examined zero-order correlations and bivariate R² values among the theoretically relevant variables to identify the pattern of associations and determine which AVA condition best characterized the data. Step 2 estimated multivariate path models to test whether DERS variables accounted for CTQ-PSS associations while controlling for overlapping predictors while also evaluating potential suppression effects and changes in predictor-outcome associations after controlling for shared variance. Step 3 used 5000 bootstrap resamples to estimate associational effects, defined as the product of the CTQ-DERS and DERS-PSS paths, with percentile 95% confidence intervals. Step 4 evaluated alternative interpretations by estimating transposed models, in which PSS variables were treated as associational variables and DERS variables as outcomes, and by testing CTQ × DERS interaction terms to rule out moderation.

Two models were estimated. Model 1 used the five CTQ subscales (Emotional Abuse, Physical Abuse, Sexual Abuse, Emotional Neglect, Physical Neglect), the DERS total score, and the PSS-10 total score. Model 2 disaggregated DERS into its six subscales (Nonacceptance of emotional responses, Difficulty engaging in goal-directed behavior, Impulse control difficulties, Lack of emotional awareness, Limited access to emotion regulation strategies, Lack of emotional clarity) and PSS-10 into Perceived Helplessness and Perceived Self-Efficacy. In line with Model 1 results, in which Physical Abuse and Physical Neglect showed no significant unique or associational effects, Model 2 retained only Emotional Abuse, Emotional Neglect, and Sexual Abuse as trauma predictors to improve parsimony. Sex was included as a predictor of CTQ subscales, and age and sex were included as covariates in the DERS and PSS equations. Path models were estimated using the lavaan package (version 0.6–18) with maximum likelihood estimation and bootstrap confidence intervals. Model fit was evaluated using the Comparative Fit Index (CFI; values > 0.90), Root Mean Square Error of Approximation (RMSEA; values < 0.06), and Standardized Root Mean Square Residual (SRMR; values < 0.08).

## Results

### Descriptive Analysis

Table 1 shows the descriptive statistics for all variables. Preliminary analyses of the contribution of demographic characteristics to the variables of interest revealed that females reported more experiences of Emotional abuse (W = 14968, *p* < .001) and Sexual abuse (W = 13639, *p* = .002) than males. In addition, females reported more Non-acceptance of emotional responses (W = 14260, *p* = .003), Impulse control difficulties (W = 14450, *p* = .001), and Limited access to emotion regulation strategies (W = 14096, *p* = .006) compared to males. Lastly, females also reported higher perceived stress overall (W = 13737, *p* = .02), specifically more Perceived helplessness (W = 13856, *p* = .01) than males. Age significantly predicted Perceived self-efficacy (t(350) = 2.26, *p* = .02), Impulse control difficulties (t(350) = -2.22, *p* = .02), and Lack of Emotional Clarity (t(350) = -4.95, *p* < .001), meaning that the older the participants the greater the sense of self-efficacy, and the lower the difficulties in these two dimensions of emotion regulation. No significant interaction effect between sex and age was observed.

**Table 1:** Descriptive statistics for variables of interest

	Total (*N* = 351)			Females (*N* = 260)			Males (*N* = 91)		
	M	SD	Range	M	SD	Range	M	SD	Range
CTQ									
Emotional Abuse	9.21	4.54	5–23	9.77	4.79	5–23	7.59	3.21	5–22
Physical Abuse	5.54	1.61	5–16	5.59	1.7	5–16	5.41	1.29	5–13
Sexual Abuse	5.84	2.37	5–24	6.01	2.6	5–24	5.34	1.41	5–16
Emotional Neglect	9.69	4.2	5–23	9.77	4.37	5–23	9.47	3.68	5–19
Physical Neglect	6.62	2.15	5–16	6.73	2.3	5–16	6.29	1.59	5–11
DERS									
Non-acceptance of emotional responses	17.12	6.15	6–30	17.69	6.17	6–30	15.48	5.81	6–30
Difficulties in goal-directed behaviors	15.83	5.04	5–25	16.16	4.93	5–25	14.88	5.27	5–24
Impulse control difficulties	13.84	5.61	6–30	14.38	5.72	6–30	12.27	5	6–26
Lack of emotional awareness	14.41	4.65	6–29	14.22	4.46	6–26	14.95	5.15	6–29
Limited access to emotion regulation strategies	22.16	7.82	8–40	22.84	7.79	8–40	20.22	7.6	9–37
Lack of emotional clarity	12.87	4.79	5–25	12.93	4.76	5–25	12.69	4.9	5–25
Total	96.22	26.37	38–164	98.23	26.38	38–164	90.49	25.63	47–147
PSS-10									
Perceived Helplessness	20.06	4.95	8–30	20.45	4.9	8–30	18.97	4.95	10–29
Perceived Self-Efficacy	12.66	2.75	6–20	12.5	2.66	6–19	13.12	2.97	7–20
Total	31.4	7.21	13–48	31.94	7.08	13–48	29.85	7.38	16–44

Note. *CTQ*=Childhood Trauma Questionnaire; *DERS*=Difficulty in Emotion Regulation Scale; *PSS-10* = Perceived Stress Scale

### Associational Variable Analysis

#### Model 1: Total Scores (CTQ Subscales, DERS Total, PSS Total)

Following the AVA framework (Weems, 2025), Step 1 examined bivariate associations among all CTQ subscales, DERS total, and PSS total (see Supplementary Table S1 for the full correlation and R² matrix). All variables were significantly intercorrelated (all *p* < .05), satisfying Condition 1. The strongest bivariate associations with DERS and PSS were observed for Emotional Neglect (*r* = .38 and 0.31) and Emotional Abuse (*r* = .36 and 0.36). DERS and PSS were strongly correlated (*r* = .64).

**Fig. 1:**
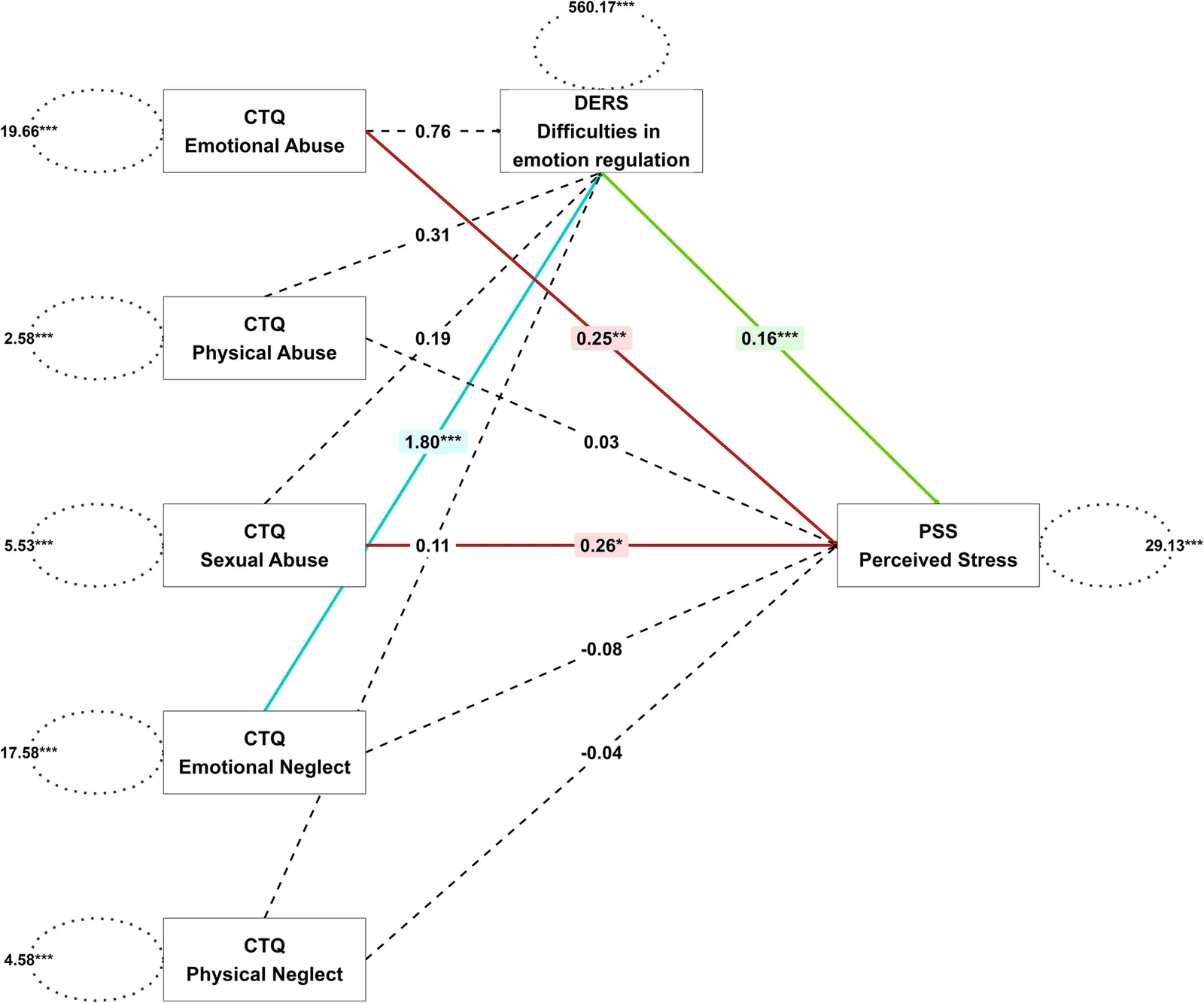
Path model of the associational variable analysis (Model 1) examining childhood trauma (CTQ) subscales, total emotion dysregulation (DERS), and total perceived stress (PSS). *Note*. CTQ=Childhood Trauma Questionnaire; DERS=Difficulty in Emotion Regulation Scale; PSS-10 = Perceived Stress Scale.Unstandardized coefficients are shown on each path. Solid lines indicate statistically significant paths (*p*<.05); dashed lines indicate non-significant paths. Light blue paths represent CTQ-to-DERS associations (a paths), green represents the DERS-to-PSS association (b path), and red represents direct CTQ-to-PSS associations controlling for DERS (c paths). Dotted grey loops denote residual variances. Age and sex were included as covariates but are omitted from the figure for clarity

In Step 2, a path model was estimated using ML with 5000 bootstrap resamples (Fig. 1), controlling for age and sex. The model showed an adequate fit (χ²(5) = 20.854, *p* = .001; CFI = 0.981; SRMR = 0.040; RMSEA = 0.095) and explained 20.1% of the variance in DERS and 44.1% in PSS. Only Emotional Neglect showed a significant association with DERS (β = 0.284, *p* < .001). When controlling for DERS, Emotional Abuse (β = 0.158, *p* = .005) and Sexual Abuse (β = 0.085, *p* = .022) retained direct associations with PSS, whereas Emotional Neglect (*r* = .31) dropped to nonsignificance (β = −0.048, *p* = .452), indicating that the association was fully statistically accounted for by DERS. No suppressor effects were observed, that is, no coefficients increased in magnitude or reversed direction when other predictors were controlled. Full path coefficients are reported in Supplementary Table S3.

In Step 3, the bootstrapped associational effect via DERS was significant for Emotional Neglect (β = 0.168, 95% CI [0.146, 0.443]), but not for Emotional Abuse (β = 0.077, 95% CI [− 0.013, 0.263]). Total effects were significant for Emotional Abuse (β = 0.234, *p* < .001) and Sexual Abuse (β = 0.095, *p* = .021).

In Step 4a, the transposed model (PSS as the associational variable) produced identical fit indices, confirming that cross-sectional data cannot distinguish between directional arrangements (Weems, 2025). In Step 4b, no CTQ × DERS interactions significantly predicted PSS (all *p* > .11), providing no evidence for moderation effects in this model specification.

#### Model 2: Subscales (CTQ Subscales, DERS Subscales, PSS Subscales)

Model 2 examined whether specific DERS subscales accounted for associations between the trauma subtypes retained from Model 1 (Emotional Abuse, Emotional Neglect, and Sexual Abuse) and the two PSS outcomes (Perceived Helplessness and Perceived Self-Efficacy). This extends the AVA framework to a subscale-level specification that involves multiple associational variables and multiple outcomes, and Weems (2025) pointed out that more complex models require additional considerations.

Step 1 confirmed Condition 1. Nearly all zero-order associations were significant (all *p* < .05), except for Sexual Abuse with Emotional Awareness (*r* = .01, *p* = .91) and Emotional Clarity (*r* = .07, *p* = .20). The strongest CTQ–DERS bivariate associations involved Emotional Abuse and Emotional Neglect with Limited access to emotion regulation strategies (*r* = .38 and 0.37) and Impulse control difficulties (*r* = .33 and 0.30) (see Supplementary Table S2).

**Fig. 2:**
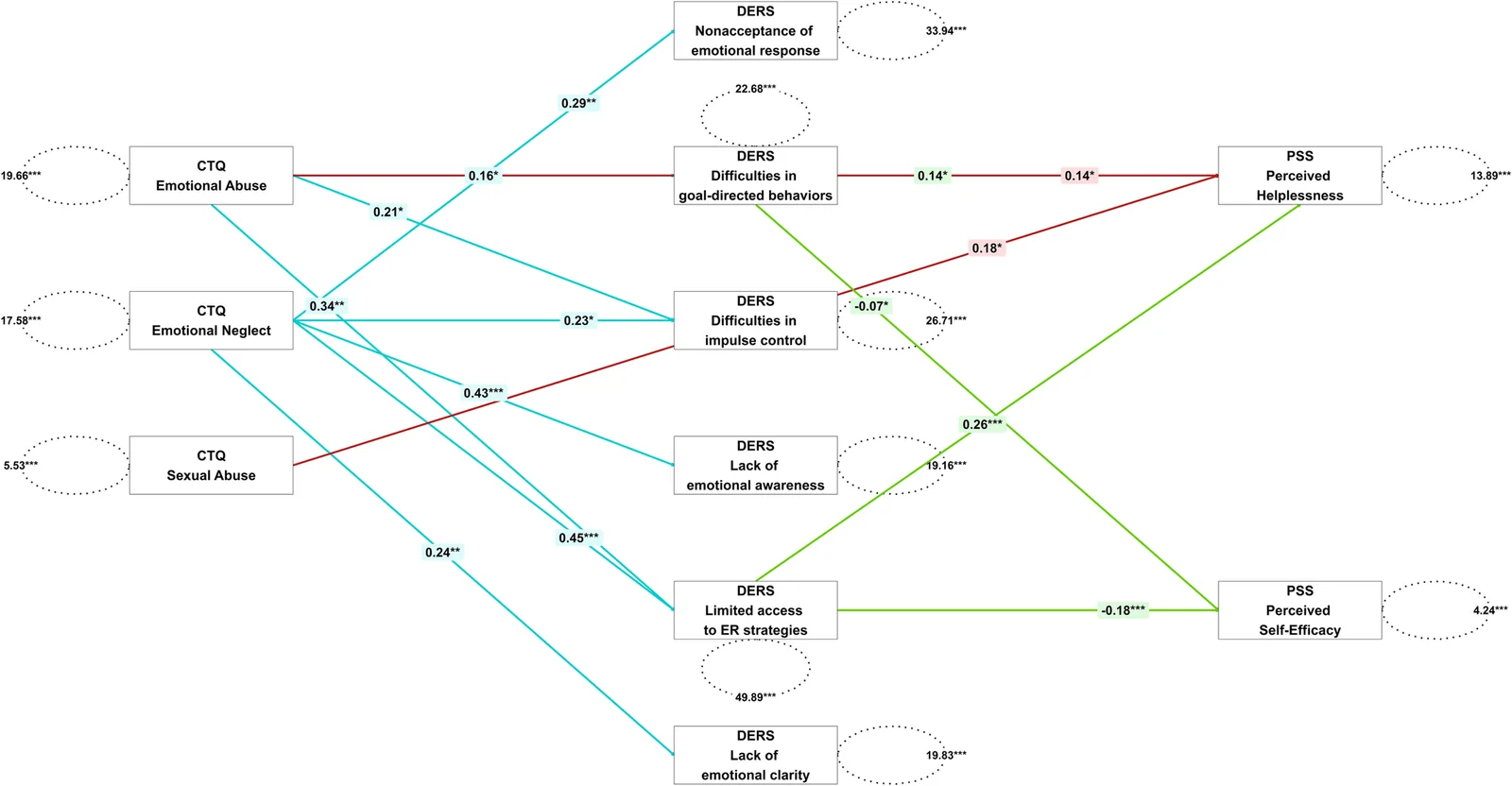
Path model of the subscale-level associational variable analysis (Model 2) examining childhood trauma (CTQ) subscales, emotion dysregulation (DERS) subscales, and perceived stress (PSS) subscales. *Note*. Only statistically significant paths (*p*<.05) are displayed. Unstandardized coefficients are shown on each path. Light blue paths represent CTQ-to-DERS associations, green paths represent DERS-to-PSS associations, and red paths represent direct CTQ-to-PSS associations controlling for DERS subscales. Dotted grey loops denote residual variances. Age and sex were included as covariates but are omitted from the figure for clarity

Steps 2 and 3 were conducted using a subscale-level path model (Fig. 2) with ML and 5000 bootstrap resamples. The model showed excellent fit (χ²(3) = 3.723, *p* = .293; CFI = 1.000; SRMR = 0.014; RMSEA = 0.026) and explained 43.4% of the variance in Perceived Helplessness and 44.3% in Perceived Self-Efficacy. Significant associational effects via DERS emerged for Emotional Abuse and Emotional Neglect with both PSS outcomes, Helplessness (β = 0.127, 95% CI [0.047, 0.231] and β = 0.127, 95% CI [0.044, 0.260]) and Self-Efficacy (β = −0.097, 95% CI [− 0.107, − 0.011] and β = −0.172, 95% CI [− 0.172, − 0.060]). Sexual Abuse showed no significant associational effects via DERS. Emotional Abuse retained a direct association with Helplessness (β = 0.129, *p* = .018), indicating partial accounting. Sexual Abuse also retained a direct association with Helplessness (β = 0.088, *p* = .028) independent of emotion regulation difficulties. By contrast, Emotional Neglect’s direct paths were nonsignificant for both outcomes, indicating full accounting by DERS. Among the DERS subscales, Limited access to emotion regulation strategies showed the strongest associations with both Helplessness (β = 0.410, *p* < .001) and Self-Efficacy (β = −0.498, *p* < .001), followed by Difficulty engaging in goal-directed behavior (β = 0.145 and − 0.134, ps < 0.05). No suppressor effects were observed. Full path coefficients are reported in Supplementary Table S4.

In Step 4a, the transposed model again produced an equivalent fit, with Emotional Neglect retaining robust direct associations with Emotional Awareness (β = 0.329), Limited access to emotion regulation strategies (β = 0.161), and Lack of emotional clarity (β = 0.163; all *p* < .02). As in Model 1, this equivalence underscores that the present cross-sectional data cannot determine directionality among these variables. In Step 4b, one significant interaction out of 36 tests (Emotional Neglect × Limited access to strategies on Helplessness, *p* = .022) was consistent with chance, and did not support a moderation reinterpretation.

## Discussion

Our study examined how childhood maltreatment is associated with perceived stress in adulthood and whether difficulties in emotion regulation account for shared variance in these associations, while controlling for sex and age. In line with the Associational Variable Analysis (AVA) framework (Weems, 2025), we interpret DERS as an associational construct rather than as a directional mediator.

Preliminary analyses revealed meaningful sex and age differences. Women reported more emotional and sexual abuse, greater difficulties in nonacceptance, impulse control, and access to regulation strategies, and higher perceived helplessness. Age was associated with higher perceived self-efficacy and fewer impulse-control and clarity difficulties. These patterns supported the use of path models to examine the relationships among trauma, emotion regulation difficulties, and perceived stress in our data.

The AVA results clarify the role of emotion regulation difficulties in the link between childhood maltreatment and perceived stress in adulthood. At the total-score level, emotional neglect was the only trauma subtype uniquely associated with DERS total. Once DERS was included, the emotional neglect–PSS association was reduced to nonsignificance, indicating that emotion dysregulation fully accounted for this association statistically. By contrast, emotional abuse and sexual abuse maintained significant direct associations with PSS, suggesting their links with adult stress perception were not fully accounted for by general emotion regulation difficulties. Importantly, the transposed model showed identical fit, meaning that the data cannot determine directionality among these variables. These findings should therefore be interpreted as evidence of robust shared variance rather than causal mediation. The subscale-level model clarified which regulatory difficulties were most relevant. DERS subscales significantly accounted for the associations of emotional abuse and emotional neglect with both perceived helplessness and perceived self-efficacy, whereas sexual abuse showed no significant associational effects via DERS. Limited access to emotion regulation strategies showed the strongest associations with both outcomes, followed by difficulty engaging in goal-directed behavior, suggesting that these two facets may be particularly relevant to stress perception in individuals who experienced emotional maltreatment. Other DERS facets did not retain significant unique associations when modeled simultaneously, suggesting that their associations overlap with those of strategy access and goal-directed behavior.

These findings are consistent with developmental models suggesting that emotionally abusive or neglectful environments interfere with the acquisition of adaptive regulation skills (Brenning & Braet, 2013; Mikulincer & Shaver, 2019). Emotionally neglectful contexts may fail to model, validate, and scaffold emotional experience, whereas emotional abuse may actively undermine emotional security and self-concept through criticism, rejection, or hostility (Greenman et al., 2024; Toof et al., 2020). Over time, these experiences may be associated with difficulties identifying effective regulatory options and maintaining goal-directed behavior during distress, which may in turn be linked to heightened perceived helplessness and reduced self-efficacy. In line with the learned helplessness model (Miller & Norman, 1979), organisms exposed to a situation where they experience unavoidable harmful stimuli (e.g., unavoidable shock) may fail to avoid such stimuli in the future when escape becomes possible (i.e., they learn “helplessness”). A revised approach proposed by Boddez and colleagues (2022) suggests that rather than learning passivity, organisms may learn controllability (Boddez et al., 2022; Duda & Joormann, 2022), meaning that prior experience with controllability could shape the perception of control later (Ly et al., 2019). Extending this mechanism would then result in a generalization of the sense of helplessness learned during unavoidable and uncontrollable maltreatment situations, reducing the perception of possible control over circumstances and increasing perceived stress (Duda & Joormann, 2022; Lieder et al., 2013). There is evidence supporting integrated control beliefs, which are related to how children learn to cope with stress (Compas et al., 1991; Jenzer et al., 2019), and several studies highlighted that an altered perception of control may contribute to the development of affective disorders and the exacerbation of perceived stress (Gallagher et al., 2014; Ly et al., 2019; McEwen & Akil, 2020; Schweizer et al., 2020). This perspective can improve our understanding of the link between trauma, emotion regulation, and stress (Weems & Silverman, 2006) and is particularly relevant when considering the consequences of exaggerated and prolonged emotional reactions to stressful situations. Under such conditions, stress can contribute to the development of various types of psychopathology (Slavich 2016a, b), and the ability to cope effectively with stress can help prevent negative consequences for mental well-being (Dijkstra & Homan, 2016). For example, individuals who do not feel competent to avoid threatening situations (low self-efficacy) may experience anxiety (Weems & Silverman, 2006). Similarly, the perception of self as having an insufficient ability to cope with situations and not having control over social situations (high helplessness) is associated with higher degrees of social anxiety (Thomasson & Psouni, 2010). This also corroborates the findings of a recent study that investigated the dynamics of coping behavior and psychological distress across different phases of the COVID-19 pandemic within an adult general population sample and revealed that maladaptive coping strategies were consistently linked to increased psychological distress, whereas adaptive strategies were associated with decreased psychological distress (Schurr et al., 2024). However, because the present data are cross-sectional, this developmental account remains a theoretically grounded interpretation rather than a causal conclusion.

A notable pattern was the stronger and more consistent role of emotional maltreatment compared with physical maltreatment. Physical abuse and physical neglect did not show meaningful associations in the multivariate AVA models once emotional abuse, emotional neglect, and other predictors were considered. This does not imply that physical maltreatment is unimportant, rather that the links with emotion regulation difficulties and perceived stress were most clearly carried by emotional abuse and emotional neglect. This pattern aligns with evidence that emotional maltreatment may be as harmful, or even more so, than physical forms of abuse because it is often chronic, relational, and embedded in the caregiving environment (Dye, 2020; Spinazzola et al., 2014). Emotional trauma has been theorized to disrupt the development of core self-regulatory capacities (Warmingham et al., 2023). Emotionally neglectful environments fail to model and validate emotional experiences, impeding the child’s ability to recognize, understand, and manage emotions (Eilert & Buchheim, 2023). Also, emotional abuse, through denigration, hostility, or rejection, may actively distort a child’s self-concept and emotional security (Baugh et al., 2019). Furthermore, physical abuse typically occurs in episodes and receives external support, while emotional trauma usually manifests chronically and subtly, making it more challenging to detect and report (Stoltenborgh et al., 2012).

Sex differences observed in the descriptive analyses further indicate that demographic variation should be considered when studying childhood maltreatment, emotion regulation, and stress (Prevoo et al., 2017). Women reported higher emotional and sexual abuse, greater difficulties in several emotion regulation domains, and greater perceived helplessness. Although sex was included as a covariate in the AVA models, the present study was not designed to test sex-specific pathways. Future work should examine whether the associations among maltreatment, regulatory difficulties, and perceived stress differ across sex or gender groups, particularly given evidence that trauma-related symptoms may be expressed differently across individuals (Cutler & Nolen-Hoeksema, 1991; Hanson et al., 2008).

Our findings align with transdiagnostic frameworks such as the Research Domain Criteria (RDoC) (Cuthbert & Insel, 2013; Fernandez et al., 2016). Within the RDoC framework, emotion regulation processes are situated at the intersection of the negative valence systems (e.g., heightened threat sensitivity in maltreated individuals) and cognitive control systems (e.g., goal-directed behavior under emotional strain), and the DERS dimensions identified in our models map directly onto these domains. Emotional maltreatment may therefore be linked to adult stress not only through heightened negative affect, but also through difficulties deploying regulatory strategies and sustaining goal-directed behavior under emotional load. This supports the view of emotion regulation difficulties as a transdiagnostic mechanism linking adversity to stress-related psychopathology (Aldao, 2016; Fernandez et al., 2016; Morawetz et al., 2024), while the AVA results clarify that these processes should be described as associational in the present cross-sectional design.

### Limitations and Future Directions

Our findings should be interpreted in light of some limitations. The cross-sectional design limits the ability to draw inferences about directionality and causality among variables. Furthermore, results may have been influenced by social desirability bias in the use of self-report measures. Importantly, our sample consisted of university students at Innsbruck University, drawn predominantly from health-related and humanities programs, and these findings were based on a restricted age range and may look different if individuals in middle or older adulthood were included, limiting generalizability to broader populations. Also, data on race/ethnicity and socioeconomic status were not collected, which further limits the generalizability of the findings. The influence of pre-existing traits (e.g., personality, anxiety trait, self-compassion) should also be explored to understand their possible role in the observed associations (Barlow et al., 2017). Additionally, our study did not assess adverse experiences in adulthood, and it remains possible that the observed associations partly reflect ongoing or more proximal stressors rather than childhood maltreatment specifically. Future studies should include measures of recent adult adversity to determine the specificity of these associations to early-life experiences.

It is important to note that we did not consider participants’ experience with psychotherapy or interventions, which could significantly affect the relationships we observed (Cloitre et al., 2019). Treatments can specifically target the underlying emotional dysregulation and biological stress responses that connect childhood adversity to later psychopathology (Cloitre et al., 2019; Miu et al., 2022). Various therapeutic modalities, such as trauma-focused therapies and somatic interventions, aim to restore autonomic flexibility and help individuals transition from maladaptive to adaptive emotion regulation strategies (Kuhfuß et al., 2021; Payne et al., 2015). Therefore, an individual’s therapeutic history may significantly influence the outcomes within this model.

Future studies should benefit from using longitudinal, multi-method designs to investigate the specific ways in which maltreatment interferes with the acquisition of healthy stress responses. Evidence suggests that the characteristics of maltreatment may have different implications for specific coping and emotion regulation strategies (Milojevich et al., 2019). Therefore, studies may also explore various forms of emotion regulation to deepen their significance and associations with psychological consequences, as well as collect details on maltreatment histories (e.g., type, timing, duration) using reliable and valid measures.

## Conclusion

These findings highlight emotion regulation difficulties as an important associational construct linking childhood emotional maltreatment and perceived stress in adulthood. Emotional abuse and emotional neglect were consistently associated with greater regulatory difficulties and higher perceived stress, with limited access to emotion regulation strategies and difficulties engaging in goal-directed behavior emerging as the most relevant regulatory dimensions. Although the cross-sectional design limits conclusions about causality or mediation, these emotion regulation domains may be relevant intervention targets for individuals with a history of childhood maltreatment. Interventions aimed at enhancing access to adaptive regulation strategies and supporting goal-directed functioning during emotional distress may be particularly relevant for improving stress-related outcomes. Future longitudinal and experimental studies are needed to clarify the temporal and causal relationships among these dimensions.

## Supplementary Information

Below is the link to the electronic supplementary material.

Supplementary Material 1

## Data Availability

https://osf.io/6z8dq/.

## Declarations

### AI Statement

During the preparation of this manuscript, the authors used Grammarly and Claude (Anthropic) for assistance with language editing and preparation of the path diagram code. All statistical analyses and interpretations were made by the authors. The authors reviewed and take full responsibility for all content in the manuscript.
