# Supplementary material for: Emotion Regulation Difficulties Account for the Associations Between Childhood Maltreatment and Perceived Stress in Young Adulthood
Source: J Child Adolesc Trauma. 2026 Jul 21;19(4):2395–407. doi: 10.1007/s40653-026-00946-8 (PMC13737239; doi:10.1007/s40653-026-00946-8)
Supplement: Supplementary file 1 [file 40653_2026_946_MOESM1_ESM.docx]

**Table S1**

*Bivariate Correlations and R² Values for Model 1 Variables (CTQ Subscales, DERS Total, PSS Total)*

| **Variable** | **1** | **2** | **3** | **4** | **5** | **6** | **7** |
| --- | --- | --- | --- | --- | --- | --- | --- |
| 1. Emotional Abuse | — | 0.233 | 0.100 | 0.487 | 0.278 | 0.131 | 0.132 |
| 2. Physical Abuse | .48^**^ | — | 0.017 | 0.133 | 0.111 | 0.030 | 0.030 |
| 3. Sexual Abuse | .32^**^ | .13^*^ | — | 0.075 | 0.049 | 0.022 | 0.044 |
| 4. Emotional Neglect | .70^**^ | .37^**^ | .27^**^ | — | 0.349 | 0.146 | 0.094 |
| 5. Physical Neglect | .53^**^ | .33^**^ | .22^**^ | .59^**^ | — | 0.057 | 0.042 |
| 6. DERS Total | .36^**^ | .17^**^ | .15^**^ | .38^**^ | .24^**^ | — | 0.411 |
| 7. PSS Total | .36^**^ | .17^**^ | .21^**^ | .31^**^ | .20^**^ | .64^**^ | — |

*Note.* Pearson correlations are presented below the diagonal; bivariate R² values are presented above the diagonal.

^*^p < .05. ^**^p < .01.

**Table S2**

*Bivariate Correlations and R² Values for Model 2 Variables (CTQ Subscales, DERS Subscales, PSS Subscales)*

| **Variable** | **1** | **2** | **3** | **4** | **5** | **6** | **7** | **8** | **9** | **10** | **11** |
| --- | --- | --- | --- | --- | --- | --- | --- | --- | --- | --- | --- |
| 1. Emotional Abuse | — | 0.487 | 0.100 | 0.069 | 0.083 | 0.110 | 0.020 | 0.142 | 0.045 | 0.123 | 0.104 |
| 2. Emotional Neglect | .70^**^ | — | 0.075 | 0.072 | 0.073 | 0.091 | 0.085 | 0.136 | 0.057 | 0.077 | 0.093 |
| 3. Sexual Abuse | .32^**^ | .27^**^ | — | 0.014 | 0.033 | 0.032 | 0.000 | 0.014 | 0.005 | 0.043 | 0.031 |
| 4. Nonacceptance | .26^**^ | .27^**^ | .12^*^ | — | 0.203 | 0.241 | 0.189 | 0.475 | 0.224 | 0.186 | 0.166 |
| 5. Goals | .29^**^ | .27^**^ | .18^**^ | .45^**^ | — | 0.378 | 0.052 | 0.499 | 0.131 | 0.285 | 0.261 |
| 6. Impulse | .33^**^ | .30^**^ | .18^**^ | .49^**^ | .61^**^ | — | 0.101 | 0.427 | 0.261 | 0.240 | 0.194 |
| 7. Awareness | .14^**^ | .29^**^ | .01 | .43^**^ | .23^**^ | .32^**^ | — | 0.170 | 0.351 | 0.047 | 0.122 |
| 8. Strategies | .38^**^ | .37^**^ | .12^*^ | .69^**^ | .71^**^ | .65^**^ | .41^**^ | — | 0.245 | 0.379 | 0.394 |
| 9. Clarity | .21^**^ | .24^**^ | .07 | .47^**^ | .36^**^ | .51^**^ | .59^**^ | .49^**^ | — | 0.137 | 0.163 |
| 10. Helplessness | .35^**^ | .28^**^ | .21^**^ | .43^**^ | .53^**^ | .49^**^ | .22^**^ | .62^**^ | .37^**^ | — | 0.532 |
| 11. Self-Efficacy | −.32^**^ | −.30^**^ | −.18^**^ | −.41^**^ | −.51^**^ | −.44^**^ | −.35^**^ | −.63^**^ | −.40^**^ | −.73^**^ | — |

*Note.* Pearson correlations are presented below the diagonal; bivariate R² values are presented above the diagonal. Nonacceptance = Nonacceptance of emotional responses; Goals = Difficulty engaging in goal-directed behavior; Impulse = Impulse control difficulties; Awareness = Lack of emotional awareness; Strategies = Limited access to emotion regulation strategies; Clarity = Lack of emotional clarity; Helplessness = Perceived Helplessness; Self-Efficacy = Perceived Self-Efficacy.

^*^p < .05. ^**^p < .01.

**Table S3**

*Path Coefficients for Model 1: Total-Score Associational Variable Analysis*

| **Path** | **B** | **SE** | *β* | *p* | **95% CI** |
| --- | --- | --- | --- | --- | --- |
| **CTQ → DERS Total** | | | | | |
| EA → DERS (a1) | 0.759 | 0.432 | .130 | .079 | [−0.076, 1.619] |
| PA → DERS (a2) | 0.309 | 0.787 | .019 | .694 | [−1.352, 1.753] |
| SA → DERS (a3) | 0.185 | 0.647 | .017 | .774 | [−0.849, 1.700] |
| EN → DERS (a4) | 1.795 | 0.452 | .284 | < .001 | [0.905, 2.707] |
| PN → DERS (a5) | 0.110 | 0.734 | .009 | .881 | [−1.424, 1.504] |
| **DERS Total → PSS Total** | | | | | |
| DERS → PSS (b) | 0.161 | 0.012 | .592 | < .001 | [0.139, 0.185] |
| **Direct CTQ → PSS (controlling for DERS)** | | | | | |
| EA → PSS (c1) | 0.251 | 0.089 | .158 | .005 | [0.076, 0.428] |
| PA → PSS (c2) | 0.033 | 0.218 | .007 | .881 | [−0.393, 0.474] |
| SA → PSS (c3) | 0.259 | 0.113 | .085 | .022 | [0.034, 0.493] |
| EN → PSS (c4) | −0.083 | 0.110 | −.048 | .452 | [−0.298, 0.143] |
| PN → PSS (c5) | −0.041 | 0.171 | −.012 | .810 | [−0.396, 0.272] |
| **Associational Effects (via DERS)** | | | | | |
| EA → DERS → PSS | 0.122 | 0.071 | .077 | .083 | [−0.013, 0.263] |
| PA → DERS → PSS | 0.050 | 0.128 | .011 | .696 | [−0.220, 0.288] |
| SA → DERS → PSS | 0.030 | 0.105 | .010 | .777 | [−0.136, 0.276] |
| EN → DERS → PSS | 0.290 | 0.076 | .168 | < .001 | [0.146, 0.443] |
| PN → DERS → PSS | 0.018 | 0.119 | .005 | .881 | [−0.230, 0.244] |
| **Total Effects** | | | | | |
| EA → PSS (total) | 0.373 | 0.105 | .234 | < .001 | [0.169, 0.586] |
| PA → PSS (total) | 0.083 | 0.244 | .018 | .735 | [−0.420, 0.555] |
| SA → PSS (total) | 0.289 | 0.125 | .095 | .021 | [0.077, 0.563] |
| EN → PSS (total) | 0.207 | 0.133 | .120 | .119 | [−0.048, 0.478] |
| PN → PSS (total) | −0.023 | 0.210 | −.007 | .911 | [−0.463, 0.366] |

*Note.* B = unstandardized coefficient; SE = standard error; β = standardized coefficient. 95% CI = percentile bootstrap confidence interval (5000 resamples) for unstandardized estimates. EA = Emotional Abuse; PA = Physical Abuse; SA = Sexual Abuse; EN = Emotional Neglect; PN = Physical Neglect.

**Table S4**

*Path Coefficients for Model 2: Subscale-Level Associational Variable Analysis*

| **Path** | **B** | **SE** | *β* | *p* | **95% CI** |
| --- | --- | --- | --- | --- | --- |
| **CTQ → DERS Subscales** | | | | | |
| EA → Nonacceptance (c1) | 0.124 | 0.101 | .091 | .219 | [−0.078, 0.319] |
| EA → Goal (c4) | 0.164 | 0.077 | .147 | .033 | [0.012, 0.312] |
| EA → Impulse (c7) | 0.214 | 0.103 | .172 | .038 | [0.004, 0.415] |
| EA → Awareness (c10) | −0.098 | 0.081 | −.095 | .229 | [−0.258, 0.062] |
| EA → Strategies (c13) | 0.338 | 0.107 | .195 | .002 | [0.128, 0.548] |
| EA → Clarity (c16) | 0.081 | 0.080 | .076 | .313 | [−0.080, 0.234] |
| EN → Nonacceptance (c2) | 0.292 | 0.103 | .199 | .005 | [0.085, 0.496] |
| EN → Goal (c5) | 0.175 | 0.091 | .145 | .056 | [−0.005, 0.353] |
| EN → Impulse (c8) | 0.228 | 0.108 | .170 | .035 | [0.018, 0.445] |
| EN → Awareness (c11) | 0.429 | 0.081 | .386 | < .001 | [0.271, 0.591] |
| EN → Strategies (c14) | 0.453 | 0.122 | .243 | < .001 | [0.211, 0.694] |
| EN → Clarity (c17) | 0.245 | 0.081 | .213 | .003 | [0.083, 0.404] |
| SA → Nonacceptance (c3) | 0.051 | 0.152 | .020 | .736 | [−0.217, 0.379] |
| SA → Goal (c6) | 0.188 | 0.110 | .088 | .087 | [−0.005, 0.429] |
| SA → Impulse (c9) | 0.152 | 0.146 | .064 | .300 | [−0.090, 0.485] |
| SA → Awareness (c12) | −0.120 | 0.099 | −.061 | .225 | [−0.287, 0.110] |
| SA → Strategies (c15) | −0.069 | 0.173 | −.021 | .690 | [−0.352, 0.328] |
| SA → Clarity (c18) | −0.019 | 0.125 | −.009 | .880 | [−0.229, 0.257] |
| **DERS Subscales → Perceived Helplessness** | | | | | |
| Nonacceptance → Help (g1) | 0.004 | 0.049 | .005 | .938 | [−0.093, 0.100] |
| Goal → Help (g2) | 0.143 | 0.070 | .145 | .042 | [0.004, 0.279] |
| Impulse → Help (g3) | 0.050 | 0.056 | .057 | .372 | [−0.059, 0.163] |
| Awareness → Help (g4) | −0.076 | 0.060 | −.072 | .204 | [−0.200, 0.040] |
| Strategies → Help (g5) | 0.259 | 0.055 | .410 | < .001 | [0.149, 0.363] |
| Clarity → Help (g6) | 0.115 | 0.063 | .112 | .068 | [−0.007, 0.240] |
| **DERS Subscales → Perceived Self-Efficacy** | | | | | |
| Nonacceptance → SE (e1) | 0.041 | 0.026 | .090 | .120 | [−0.010, 0.091] |
| Goal → SE (e2) | −0.073 | 0.037 | −.134 | .046 | [−0.143, −0.001] |
| Impulse → SE (e3) | 0.020 | 0.031 | .041 | .519 | [−0.041, 0.081] |
| Awareness → SE (e4) | −0.063 | 0.033 | −.106 | .058 | [−0.128, 0.001] |
| Strategies → SE (e5) | −0.175 | 0.028 | −.498 | < .001 | [−0.231, −0.120] |
| Clarity → SE (e6) | −0.043 | 0.031 | −.075 | .169 | [−0.106, 0.017] |
| **Direct CTQ → Helplessness (controlling for DERS)** | | | | | |
| EA → Help (h1) | 0.141 | 0.060 | .129 | .018 | [0.026, 0.259] |
| EN → Help (h2) | −0.063 | 0.066 | −.053 | .346 | [−0.195, 0.064] |
| SA → Help (h3) | 0.183 | 0.083 | .088 | .028 | [0.022, 0.351] |
| **Direct CTQ → Self-Efficacy (controlling for DERS)** | | | | | |
| EA → SE (f1) | −0.046 | 0.035 | −.075 | .196 | [−0.113, 0.026] |
| EN → SE (f2) | −0.002 | 0.043 | −.003 | .967 | [−0.082, 0.082] |
| SA → SE (f3) | −0.091 | 0.051 | −.078 | .075 | [−0.193, 0.008] |
| **Associational Effects (via DERS subscales)** | | | | | |
| EA → DERS → Help | 0.139 | 0.047 | .127 | .003 | [0.047, 0.231] |
| EN → DERS → Help | 0.150 | 0.054 | .127 | .005 | [0.044, 0.260] |
| SA → DERS → Help | 0.024 | 0.072 | .011 | .741 | [−0.095, 0.191] |
| EA → DERS → SE | −0.059 | 0.025 | −.097 | .016 | [−0.107, −0.011] |
| EN → DERS → SE | −0.113 | 0.028 | −.172 | < .001 | [−0.172, −0.060] |
| SA → DERS → SE | 0.012 | 0.039 | .010 | .763 | [−0.078, 0.077] |
| **Total Effects** | | | | | |
| EA → Help (total) | 0.280 | 0.071 | .256 | < .001 | [0.142, 0.417] |
| EN → Help (total) | 0.088 | 0.084 | .074 | .296 | [−0.078, 0.249] |
| SA → Help (total) | 0.207 | 0.085 | .099 | .015 | [0.065, 0.395] |
| EA → SE (total) | −0.105 | 0.040 | −.173 | .008 | [−0.180, −0.024] |
| EN → SE (total) | −0.115 | 0.046 | −.175 | .012 | [−0.205, −0.025] |
| SA → SE (total) | −0.079 | 0.055 | −.068 | .148 | [−0.198, 0.019] |

*Note.* B = unstandardized coefficient; SE = standard error; β = standardized coefficient. 95% CI = percentile bootstrap confidence interval (5000 resamples) for unstandardized estimates. EA = Emotional Abuse; EN = Emotional Neglect; SA = Sexual Abuse; Help = Perceived Helplessness; SE = Perceived Self-Efficacy.
